# Variability in accuracy of prostate cancer segmentation among radiologists, urologists, and scientists

**DOI:** 10.1002/cam4.3386

**Published:** 2020-08-18

**Authors:** Michael Y. Chen, Maria A. Woodruff, Prokar Dasgupta, Nicholas J. Rukin

**Affiliations:** ^1^ Science and Engineering Faculty Queensland University of Technology Brisbane Queensland Australia; ^2^ Redcliffe Hospital Metro North Hospital and Health Service Herston Queensland Australia; ^3^ School of Medicine University of Queensland Brisbane Queensland Australia; ^4^ Herston Biofabrication Institute Metro North Hospital and Health Service Brisbane Australia; ^5^ King’s College London Guy’s Hospital London United Kingdom

**Keywords:** 3D printing, 3D model, MRI, prostate, segmentation

## Abstract

**Background:**

There is increasing research in using segmentation of prostate cancer to create a digital 3D model from magnetic resonance imaging (MRI) scans for purposes of education or surgical planning. However, the variation in segmentation of prostate cancer among users and potential inaccuracy has not been studied.

**Methods:**

Four consultant radiologists, four consultant urologists, four urology trainees, and four nonclinician segmentation scientists were asked to segment a single slice of a lateral T3 prostate tumor on MRI (“Prostate 1”), an anterior zone prostate tumor MRI (“Prostate 2”), and a kidney tumor computed tomography (CT) scan (“Kidney”). Time taken and self‐rated subjective accuracy out of a maximum score of 10 were recorded. Root mean square error, Dice coefficient, Matthews correlation coefficient, Jaccard index, specificity, and sensitivity were calculated using the radiologists as the ground truth.

**Results:**

There was high variance among the radiologists in segmentation of Prostate 1 and 2 tumors with mean Dice coefficients of 0.81 and 0.58, respectively, compared to 0.96 for the kidney tumor. Urologists and urology trainees had similar accuracy, while nonclinicians had the lowest accuracy scores for Prostate 1 and 2 tumors (0.60 and 0.47) but similar for kidney tumor (0.95). Mean sensitivity in Prostate 1 (0.63) and Prostate 2 (0.61) was lower than specificity (0.92 and 0.93) suggesting under‐segmentation of tumors in the non‐radiologist groups. Participants spent less time on the kidney tumor segmentation and self‐rated accuracy was higher than both prostate tumors.

**Conclusion:**

Segmentation of prostate cancers is more difficult than other anatomy such as kidney tumors. Less experienced participants appear to under‐segment models and underestimate the size of prostate tumors. Segmentation of prostate cancer is highly variable even among radiologists, and 3D modeling for clinical use must be performed with caution. Further work to develop a methodology to maximize segmentation accuracy is needed.

## Graphical abstract

Segmentation of prostate cancer is highly variable even among experienced radiologists and urologists. Less experienced users are likely to underestimate the size of prostate cancer tumors.

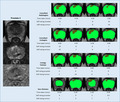

## 1 INTRODUCTION

Segmentation is a process of creating patient‐specific three‐dimensional (3D) digital models based on target anatomy in computed tomography (CT) or magnetic resonance imaging (MRI) scans. Specialized segmentation software provides tools to aid in creating a 3D digital model. This 3D model can then be further manipulated with computer‐aided design (CAD) software.

After segmentation, the model can be used for 3D modeling or 3D printing which is currently being explored in urology.^1^, ^2^ Many researchers are investigating the clinical use for 3D‐printed anatomical models of prostate cancer ^3^, ^4^, ^5^, ^6^, ^7^, ^8^, ^9^, ^10^ and kidney cancer.^11^, ^12^, ^13^, ^14^, ^15^, ^16^ In addition to 3D printing, 3D digital modeling can allow additional technology to be incorporated into service provision such as augmented reality robotic prostate surgery.^17^, ^18^

What has not been established in this field is the variability in the segmentation accuracy of these 3D prostate models. Many research groups are using 3D segmentation of prostate cancer in their research, but the segmentation methodology is usually not described in detail. Inaccurate 3D models being used in clinical practice has the potential to cause clinical harm.

**FIGURE 1:**
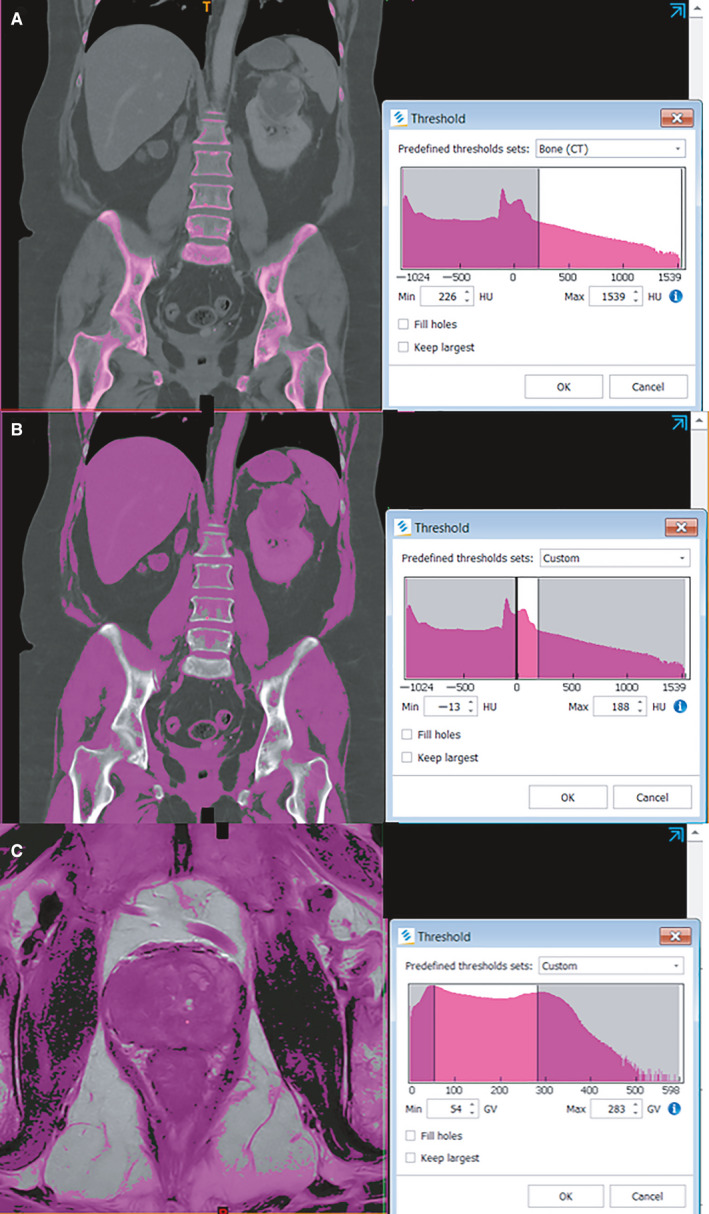
A comparison of using threshold density to initiate the segmentation process. On CT imaging of bone (A) the high density allows for rapid segmentation from surrounding soft tissue. To segment the kidney on CT (B), other soft tissues such as spleen are included but the kidney is separated from surrounding adipose tissue. To segment prostate on MRI (C) the rectum and capsular tissue are included, requiring additional manual segmentation. Screenshots taken on Mimics 21.0 (Materialise, Leuven, Belgium). The threshold for bone is predefined by the software, while the others were manually selected

Segmentation software has many tools to automate the process; however, their effectiveness depends on the target anatomy. For example, segmentation of bone is straightforward due to the high density of mineralized bone compared to soft issue, which more easily facilitates automation and even deep learning of bone segmentation.^19^, ^20^ However, this process cannot be easily applied to soft tissue in urology. Furthermore, the segmentation of prostate from surrounding soft tissue is further complicated on MRI as surrounding tissue is of similar intensity compared with kidney CT. Figure 1 highlights these differences by showing the results of density or intensity thresholds without manual segmentation. The separation of the prostate gland from surrounding soft tissue is only the first step, as the user must then separate the prostate tumor from the rest of the prostate. Interpretation of prostate cancers on MRI is known to be a challenging task which should only be performed by subspecialized radiologists.^21^, ^22^

The aim of this study is to quantify the high variability of segmentation in prostate cancer MRI while using a kidney CT as an alternative imaging technique comparison. In addition, the project aims to compare results between radiologists, urologists, urology trainees, and nonclinician researchers to show the potential inaccuracy of segmentation from less experienced users, as well as to determine trends in over‐segmenting or under‐segmenting of the tumors for less experienced users.

## 2 MATERIALS AND METHODS

### 2.1 Participants

Ethics approval was obtained from the hospital's human research ethics committee (study reference: LNR/2019/QRBW/51927) and patient imaging was used with written informed consent. Four groups were recruited which were consultant radiologists, consultant urologists, urology trainees, and scientists with segmentation experience and there were four participants in each group (Table 1).

**TABLE 1:** Summary of participants in each group recruited to the study

	Radiologists	Urologists	Urology trainees	Nonclinicians
Number of participants	4	4	4	4
Urology experience	>3 y of experience interpreting prostate MRI	>3 y after completing specialty training	1‐3 y of experience in urology	Nil
Segmentation experience	Nil	Nil	Nil	Experienced in orthopedic or vascular segmentation but not urology

### 2.2 Image selection

One prostate MRI showing a lateral T3 peripheral zone tumor was assigned as “Prostate 1” and one prostate MRI showing a small anterior zone tumor was assigned as “Prostate 2”. Scans included T2, diffusion‐weighted imaging (DWI) and apparent diffusion coefficient (ADC) views in 3‐mm slices. One kidney CT of a 6‐cm superior pole tumor was also included for comparison in 1‐mm slices.

A single slice of each scan was selected by the study author MC as the slice at which tumor diameter appeared maximal. Users could scroll through other slices during segmentation; however, they were asked to segment the single slice instead of the whole 3D model to simplify the process for the participants with no experience with the software.

### 2.3 Segmentation study

Participants were recruited and asked to perform segmentation of the three scans on the single slices specified. The software used was Mimics 21.0 (Materialise), an FDA‐approved software for 3D medical segmentation. The study author MC explained the basic tools used in this study to the participants. The radiologist report of the scans was available if needed. Nonclinicians were encouraged to also peruse other online resources for assistance if needed during the segmentation. No advanced or automated segmentation tools were used and participants used only the manual selection and eraser tools to perform the segmentation.

Participants were then asked to segment the target organ including the tumor, and then the tumor alone for Prostate 1, Prostate 2, and Kidney images. Segmentation density or intensity thresholds were set to help them separate anatomy from surrounding fat and these were identical between participants. Participants were timed by the study author MC during their segmentation with the time taken rounded to the nearest 15 seconds. Help with the software tools was provided but no help with the actual segmentation or anatomy was provided. After each image, participants were asked to self‐rate their subjective confidence in the accuracy of their segmentation of the whole organ and tumor out of a maximum score of 10.

### 2.4 Image analysis

**FIGURE 2:**
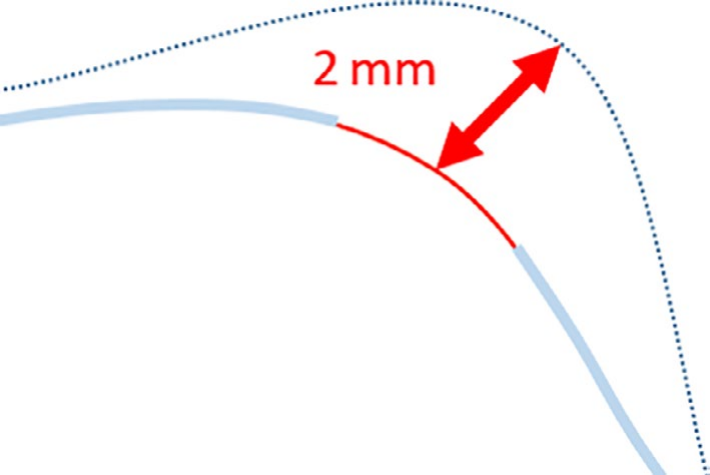
An example of how the point comparison method works to calculate the distance between two points which is then averaged across the whole model using the root mean square (RMS) calculation method

All segmented images were combined into figures for a visual comparison. To quantify the differences between individual segmentations, 3‐matic 13.0 (Materialise, Leuven, Belgium) software was used to calculate the root mean square (RMS) difference between two segmented images using a point comparison method. Figure 2 shows how the point comparison method calculates a distance between two points on each segmentation. The RMS method adjusts for both positive and negative differences to give an absolute value.

The segmentation images were converted to binary black and white images and analyzed in software MATLAB R2019b (The MathWorks Inc). Dice coefficient, Matthews correlation coefficient (MCC), Jaccard index, specificity, and sensitivity were calculated using the code developed by Thanh et al.^23^

The radiologists were used as the ground truth in segmentation comparisons with other users, and analysis was repeated for each radiologist to account for variation. For the radiologist group, their segmentation was compared to the other three radiologists.

## 3 RESULTS

### 3.1 Prostate 1

**FIGURE 3:**
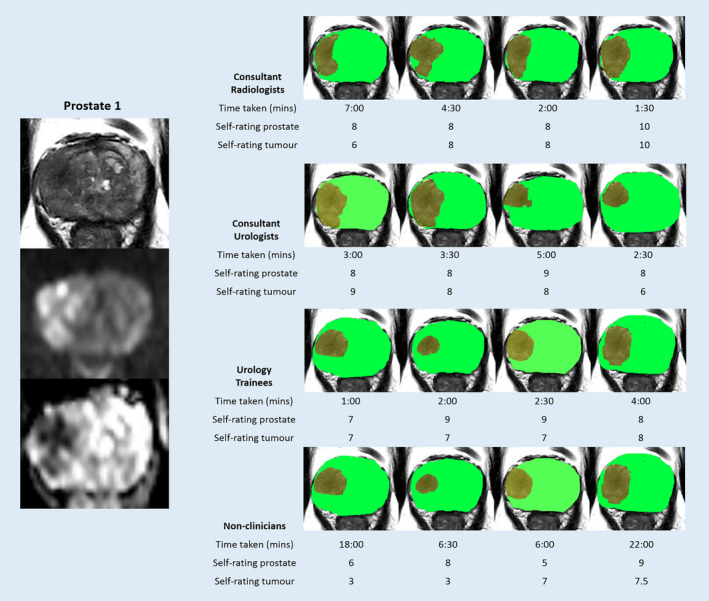
Segmentation results of Prostate 1 from 16 participants with time taken and self‐rated accuracy

Figure 3 shows the results of the segmentation of the lateral T3 prostate tumor (Prostate 1) between the 16 participants. None of the radiologists included the anterior portion of the capsule, while two participants in each of the other groups included this in their segmentation which leads to the lower specificity in those groups (Table 2). The segmentation of the tumor was more variable even among radiologists with a mean Dice coefficient of 0.81, while nonclinicians had the lowest overlap with radiologists (Dice coefficient 0.60). Urologists, urology trainees, and nonclinicians overall segmented less tumor than radiologists with a mean sensitivity combining all three groups of 0.63 compared to a combined specificity of 0.92 (Table 3). All clinicians rated their own confidence quite highly while nonclinicians were less confident particularly regarding the tumor (5.1/10) (Table 4). The mean time taken by nonclinicians was longest at 13:08 minutes compared to 3:45 minutes for the radiologists (Table 4).

**TABLE 2:** Variation in segmentation of prostate/kidney compared to the radiologist group

	Mean RMS, mm (SD)	Mean Dice coefficient (SD)	Mean MCC (SD)	Mean Jaccard index (SD)	Mean specificity (SD)	Mean sensitivity (SD)
Prostate 1						
Radiologists	0.12 (0.03)	0.97 (0.01)	0.92 (0.01)	0.95 (0.01)	—	—
Urologists	0.41 (0.21)	0.94 (0.03)	0.83 (0.08)	0.89 (0.05)	0.81 (0.12)	0.99 (0.01)
Urology trainees	0.35 (0.21)	0.95 (0.02)	0.86 (0.07)	0.91 (0.04)	0.86 (0.11)	0.98 (0.02)
Nonclinicians	0.36 (0.10)	0.95 (0.01)	0.86 (0.03)	0.90 (0.02)	0.90 (0.08)	0.95 (0.04)
Prostate 2						
Radiologists	0.19 (0.14)	0.96 (0.01)	0.91 (0.01)	0.93 (0.01)	—	—
Urologists	0.20 (0.06)	0.96 (0.01)	0.89 (0.04)	0.92 (0.03)	0.93 (0.04)	0.96 (0.01)
Urology trainees	0.26 (0.11)	0.97 (0.01)	0.91 (0.02)	0.93 (0.02)	0.94 (0.05)	0.97 (0.02)
Nonclinicians	2.10 (1.96)	0.80 (0.17)	0.71 (0.21)	0.70 (0.24)	0.98 (0.03)	0.71 (0.26)
Kidney						
Radiologists	0.27 (0.07)	0.98 (0.01)	0.95 (0.01)	0.96 (0.01)	—	—
Urologists	0.30 (0.12)	0.98 (0.01)	0.95 (0.01)	0.96 (0.01)	0.97 (0.01)	0.98 (0.01)
Urology trainees	0.26 (0.09)	0.98 (0.01)	0.96 (0.01)	0.97 (0.01)	0.98 (0.01)	0.98 (0.01)
Nonclinicians	0.38 (0.14)	0.98 (0.01)	0.96 (0.01)	0.96 (0.01)	0.96 (0.01)	0.99 (0.01)

**TABLE 3:** Variation in segmentation of tumor compared to the radiologist group

	Mean RMS, mm (SD)	Mean Dice coefficient (SD)	Mean MCC (SD)	Mean Jaccard index (SD)	Mean specificity (SD)	Mean sensitivity (SD)
Prostate 1						
Radiologists	1.15 (0.46)	0.81 (0.04)	0.73 (0.05)	0.69 (0.06)	—	—
Urologists	2.49 (1.28)	0.73 (0.10)	0.64 (0.10)	0.58 (0.12)	0.88 (0.09)	0.74 (0.22)
Urology trainees	2.50 (0.98)	0.70 (0.13)	0.62 (0.12)	0.56 (0.15)	0.93 (0.05)	0.65 (0.19)
Nonclinicians	2.65 (1.33)	0.60 (0.15)	0.56 (0.12)	0.45 (0.16)	0.96 (0.06)	0.51 (0.23)
Prostate 2						
Radiologists	3.01 (1.38)	0.58 (0.10)	0.57 (0.08)	0.41 (0.10)	—	—
Urologists	2.41 (1.32)	0.59 (0.16)	0.57 (0.14)	0.44 (0.18)	0.88 (0.08)	0.82 (0.22)
Urology trainees	3.13 (1.51)	0.54 (0.14)	0.51 (0.15)	0.39 (0.14)	0.95 (0.03)	0.56 (0.24)
Nonclinicians	3.94 (1.75)	0.47 (0.15)	0.48 (0.12)	0.32 (0.13)	0.97 (0.04)	0.44 (0.27)
Kidney						
Radiologists	0.44 (0.11)	0.96 (0.01)	0.92 (0.01)	0.92 (0.01)	—	—
Urologists	0.64 (0.16)	0.96 (0.01)	0.92 (0.02)	0.92 (0.02)	0.94 (0.03)	0.97 (0.03)
Urology trainees	0.65 (0.24)	0.95 (0.01)	0.91 (0.03)	0.91 (0.02)	0.97 (0.02)	0.94 (0.02)
Nonclinicians	0.80 (0.43)	0.95 (0.02)	0.90 (0.04)	0.90 (0.04)	0.98 (0.01)	0.92 (0.04)

**TABLE 4:** Mean self‐rating and time taken by each group of participants for the three segmented images

	Mean self‐rating of prostate/kidney from 1‐10, (SD)	Mean self‐rating of tumor from 1‐10, (SD)	Mean time taken, minutes (SD)
Prostate 1			
Radiologists	8.5 (1.0)	8.0 (1.6)	3:45 (2:32)
Urologists	8.3 (0.5)	7.8 (1.3)	3:30 (1:05)
Urology trainees	8.3 (0.9)	7.3 (0.5)	2:22 (1:15)
Nonclinicians	7.0 (1.8)	5.1 (2.5)	13:08 (8:06)
Combined	8.0 (1.1)	7.0 (1.7)	5:41 (5:54)
Prostate 2			
Radiologists	8.3 (1.3)	8.0 (1.4)	3:38 (1:11)
Urologists	8.5 (1.3)	7.3 (1.9)	4:15 (1:33)
Urology trainees	8.0 (2.0)	6.8 (2.2)	2:38 (1:30)
Nonclinicians	6.9 (2.8)	4.5 (3.0)	7:15 (2:40)
Combined	7.9 (1.7)	6.6 (2.2)	4:26 (2:24)
Kidney			
Radiologists	9.3 (1.0)	9.0 (1.4)	2:30 (1:46)
Urologists	9.5 (0.6)	8.8 (1.3)	2:41 (1:26)
Urology trainees	9.0 (0.8)	9.0 (0.8)	1:45 (1:11)
Nonclinicians	7.8 (1.0)	6.3 (3.1)	3:53 (1:26)
Combined	8.9 (1.0)	8.3 (1.9)	2:42 (1:32)

### 3.2 Prostate 2

**FIGURE 4:**
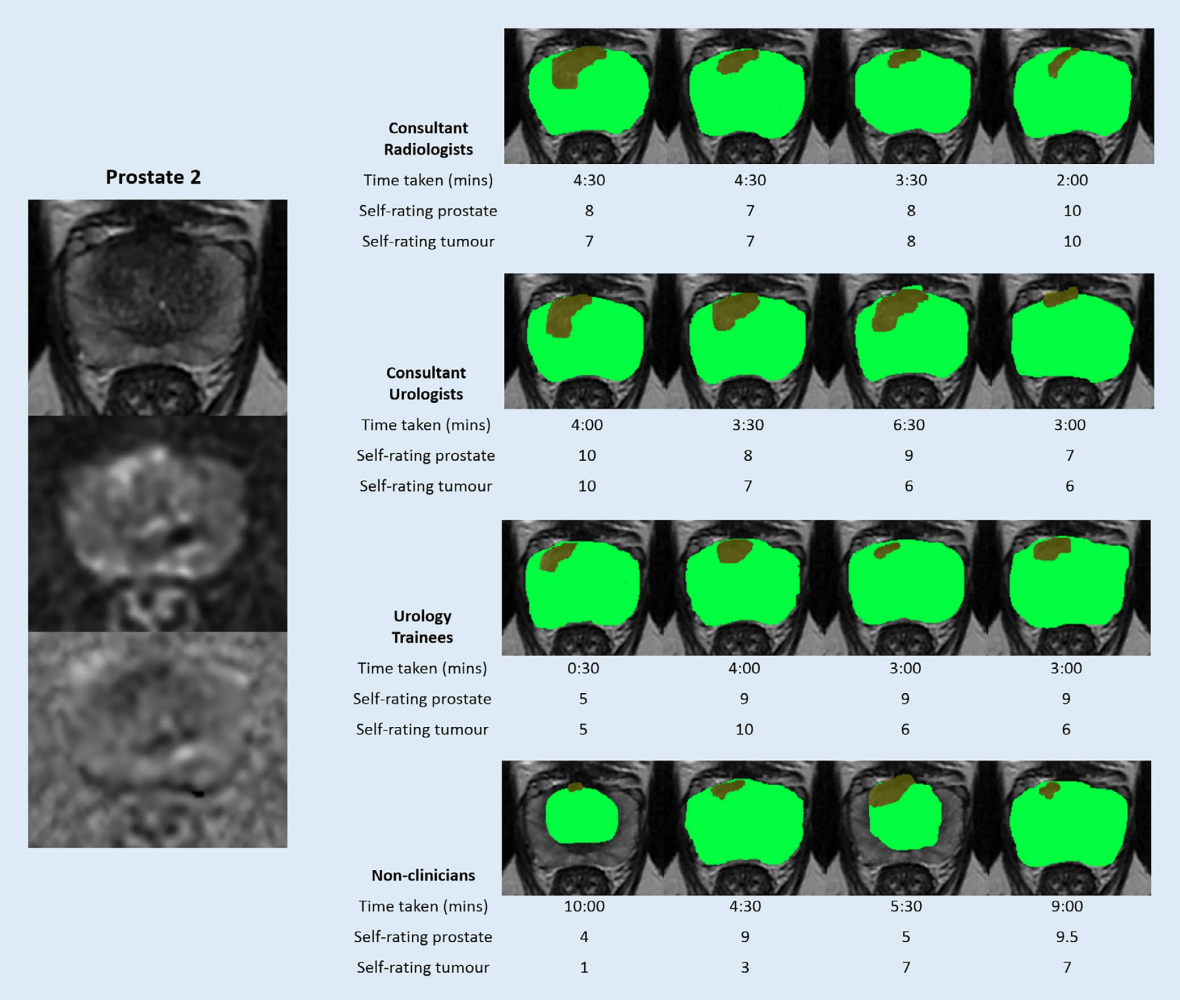
Segmentation results of Prostate 2 from 16 participants with time taken and self‐rated accuracy

Figure 4 shows the segmentation results of the smaller anterior zone prostate tumor (Prostate 2). Two of the nonclinicians segmented only the central zone of the prostate gland in this image and did not include the peripheral zone, but segmentation of the gland was otherwise consistent (Table 2). There was highly variable segmentation of the tumor between all participants including among radiologists. Intra‐group comparison among radiologists showed a mean Dice coefficient of 0.58 for the tumor compared to 0.81 in Prostate 1 (Table 3). As a result of the high variability among radiologists, mean accuracy scores were low across all other groups. Mean combined sensitivity across urologist, urology trainee, and nonclinician groups remained lower at 0.61 compared to a combined specificity of 0.93. Nonclinicians reported low confidence with a mean score of 6.9/10 and 4.5/10 for prostate and tumor, respectively, while taking a higher mean time of 7:15 minutes (Table 4).

### 3.3 Kidney

**FIGURE 5:**
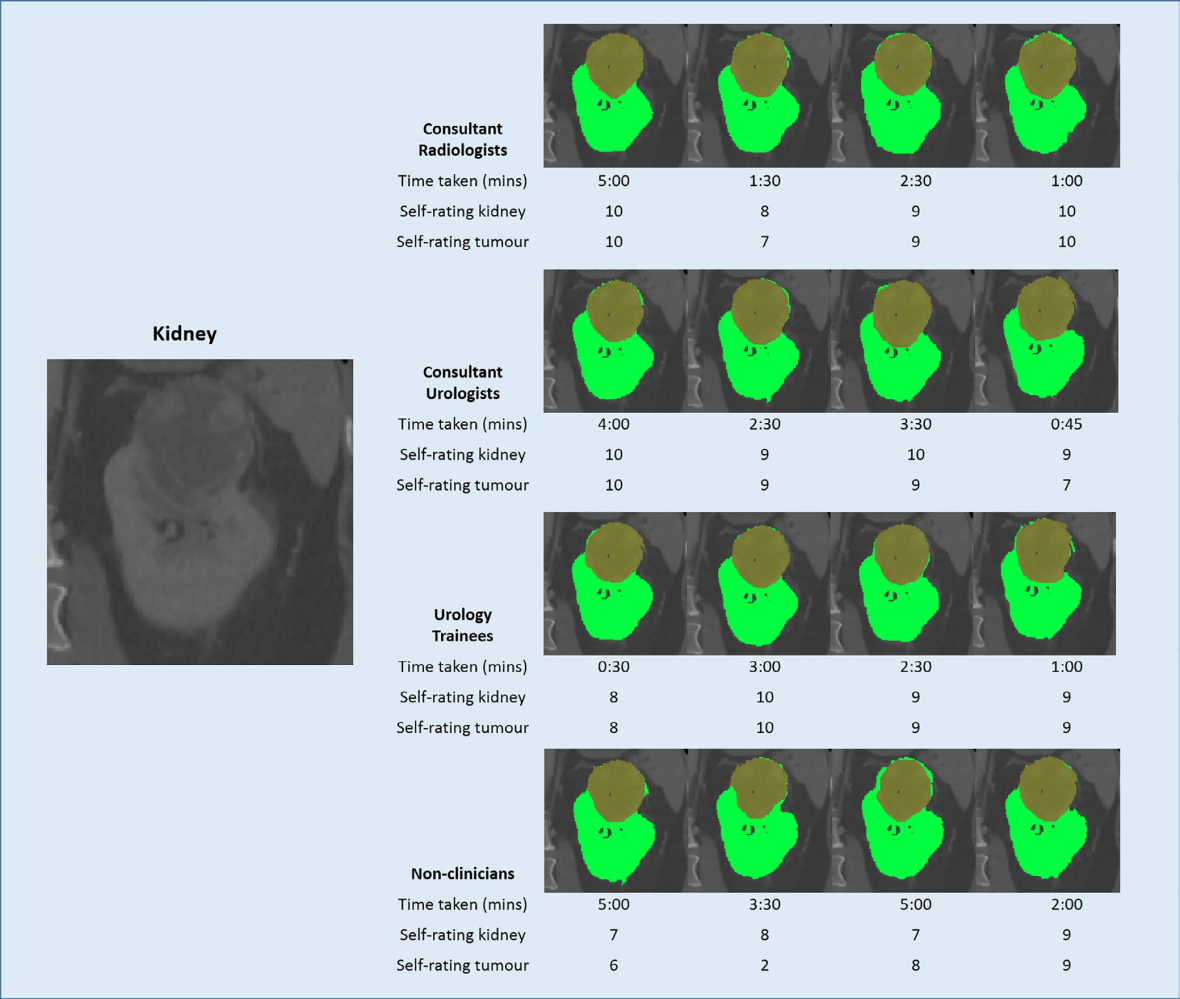
Segmentation results of Kidney from 16 participants with time taken and self‐rated accuracy

Figure 5 shows the kidney CT segmentation results. Both the kidney and tumor were very consistent across all 16 participants. Segmentation accuracy scores were high across all participants and groups without any score below 0.90. Participants performed the segmentation of this image quickly with a mean time of 2:42 minutes (Table 4). Nonclinicians took a longer mean time of 3:53 minutes. Clinicians were overall very confident but nonclinicians still had uncertainty with a mean self‐rating of the tumor of 6.3/10.

Overall, the mean self‐rating for the kidney tumor was higher than both prostate tumors (8.3 vs 7.0 vs 6.6, *P* = .04). Similarly, participants felt more confident about the segmentation of the whole kidney than Prostate 1 and Prostate 2 (8.9 vs 7.9 vs 8.0, *P* = .06).

## 4 DISCUSSION

There has been significant progress in the clinical use of segmentation in recent years used for a variety of purposes such as 3D modeling and 3D printing. Many research groups are now exploring its use in prostate cancer in a multitude of ways related to surgical planning, education, histopathological correlation, and augmented reality surgery.^3^, ^4^, ^5^, ^6^, ^7^, ^9^, ^10^, ^17^, ^24^ However, the interpretation and segmentation of prostate MRI are complicated when compared to bone or kidney tumors. These preliminary results suggest that segmentation of the prostate gland itself is fairly consistent, although nonclinicians may have more errors as one might expect. However, the segmentation of the prostate tumor is highly variable, even among consultant radiologists and urologists. The sensitivity and specificity results suggest that with less experience with prostate MRI, users will segment less of the tumor than an experienced user.

Recognizing prostate cancer on MRI is known to be challenging and relies on multiple views (T2, DWI, and ADC), which increases its complexity compared to CT.^25^ Hansen et al^21^ reviewed 158 prostate MRI scans reported by radiologists in the community and re‐evaluated by subspecialized uroradiologists and found disagreement in 54% of cases. In addition, the uroradiologists had a higher negative predictive value and positive predictive value based on MRI fusion prostate biopsy results.

Even with expert radiologist reporting, up to 25% of significant cancer may be missed when compared to the histopathology of prostate specimens.^26^, ^27^ The requirements for reporting prostate MRI vary by area, but literature suggests that analysis of 50‐100 cases is required to become proficient.^22^ Therefore, for diagnostic purposes, a prostate MRI should be interpreted by an experienced MRI radiologist, but there has been little consideration for the segmentation process of prostate cancer for using 3D models in clinical practice.

Some studies do not describe their segmentation methodology beyond stating the software used. Wake et al^16^ state that their segmentation of renal cancer was performed by a research scientist with 5 years of experience with segmentation and post‐processing. Porpiglia et al^17^ state that their prostate cancer models were segmented by bioengineers using a contour‐based method which was then digitally overlaid to facilitate augmented reality robotic prostate surgery. They conclude that collaboration between urologists, radiologists, and bioengineers is essential and comparison to 3D scanning of the prostate showed good agreement. Naturally, these researchers were closely supervised by clinicians in these studies. However, as 3D modeling and 3D printing enter mainstream use, this could foreseeably become a commercial service in the future. Clinicians less familiar with the segmentation process may overestimate the level of accuracy provided by these models and ideally a standardized methodology should be established. Researchers should include who reviewed the segmentation accuracy in their publication methodology. This preliminary study demonstrates that collaboration between radiologists, urologists, and nonclinician researchers is essential in ongoing research in 3D modeling of prostate cancer.

The slice thickness of the prostate MRI was 3 mm in our study, which is routine in clinical practice, while CT usually includes 1‐mm slices. Although it did not affect the segmentation in this study of a single slice, in our experience, smaller slice thickness is important for segmentation of 3D structures and this should be considered in segmentation research. Usually, a slice thickness of more than 3 mm is unable to be segmented effectively.

Recent research into artificial intelligence (AI), radiomics, and machine deep learning is likely to simplify the segmentation process in the future.^28^, ^29^, ^30^, ^31^, ^32^ There have been research groups examining deep learning in prostate MRI segmentation.^33^, ^34^, ^35^ This would significantly reduce the workload. It is likely that the segmentation process will one day become automated, but a radiologist should still verify the accuracy of any 3D models created by AI.

This study's limitations are its relatively small sample size and lack of a gold standard for comparison. It has been shown that the dimensions of tumors on histopathology will vary from radiological dimensions in prostate cancer^36^ as well as kidney cancer.^37^ Therefore, the collective results of the four radiologists were used as the “ground truth” rather than histopathology to quantify segmentation accuracy. The variance seen in the segmentation of the radiologists demonstrates that this is not an accurate gold standard. Particularly in the anterior zone tumor of “Prostate 2,” the high amount of variation between radiologists makes the results of the analysis difficult to interpret.

Our objective in this study was to maximize the number of participants across different groups and this meant segmentation of whole organs across multiple scans was impractical. The small number of scans included in this study is a significant limitation and reduces the generalizability of these results. However, based on the variance across all participants and the errors of the nonclinicians, it is hypothesized that there is a need for a segmentation methodology that reduces uncertainty in future use of this technology.

The two prostate tumors selected included a prominent T3 lateral tumor and a smaller anterior zone tumor. The smaller anterior zone tumor measured around 8 mm and showed more variability as expected. Very small tumors less than 5 mm are challenging to diagnose on MRI and are likely to show even greater variability in segmentation but was not included in this study.

In the study, participants segmented only the kidney and the kidney tumor to demonstrate how the tumors in the prostate are uniquely challenging to segment. However, in practice, a 3D kidney model would require inclusion of the collecting system and blood vessels to be clinically useful, and accurate segmentation of intraparenchymal segmental arteries or accurate proximity of the tumor to calyces can be very challenging. Therefore, we do not suggest that 3D kidney models are easy to segment, and issues around their accuracy in clinical practice are still very relevant.

As this segmentation was for research purposes only and not clinical use, the time taken was very low for some participants. It is possible that some of the variance is also caused by the short time taken by participants. Although the segmentation was simplified to a single image, digital literacy may also have played a part. This could explain the low variance and low time taken among the urology trainees, the youngest group of participants.

In this study, segmentation of a single slice only was performed. Segmentation of the extremes, such as the prostate apex, can be challenging and was not evaluated in this study. Segmentation of a whole 3D model is a lengthy process which requires a deeper understanding of software features and it was not feasible for all participants to have that level of expertise. At our institution, complete and accurate segmentation of a prostate and tumor can take up to 2 hours, particularly for those inexperienced in the software. Therefore, segmentation of prostate cancer may need to be performed by a scientist or engineer with segmentation software expertise. Based on these results showing significant variation in segmentation accuracy, future segmentation may need to be performed by an experienced software user in close collaboration with at least one experienced MRI uroradiologist. Use of segmentation, 3D modeling, and 3D printing is a novel field and requires a multidisciplinary approach with close collaboration between radiologists, urologists, and nonclinician researchers to ensure accuracy and clinical safety.

## 5 CONCLUSIONS

The segmentation process used to create digital 3D models of prostate cancer from MRI scans is more difficult than segmentation of other pathologies such as kidney cancer. Segmentation performed by those with less experience in prostate MRI appear to underestimate tumor size. As research in 3D printing and 3D modeling of prostate cancer continues, additional steps to ensure accurate segmentation may be needed given the variation observed even between experienced clinicians. Future studies with a larger sample size are needed to determine what measures could effectively increase the accuracy of prostate cancer segmentation to a clinically safe level.

## CONFLICT OF INTEREST

The authors wish to declare no conflict of interest.

## AUTHOR CONTRIBUTIONS

MYC and NJR conceived the study design with input from PD. MYC collected the data and conducted statistical analysis with assistance from NJR and MAW. MYC wrote the manuscript with supervision and editing from MAW, PD, and NJR.

## Data Availability

Data sharing is not applicable to this study.
